# Internal and external aspects of freedom of choice in mental health: cultural and linguistic adaptation of the Hungarian version of the Oxford CAPabilities questionnaire—Mental Health (OxCAP-MH)

**DOI:** 10.1186/s40359-021-00660-0

**Published:** 2021-10-18

**Authors:** Timea Mariann Helter, Ildiko Kovacs, Andor Kanka, Orsolya Varga, Janos Kalman, Judit Simon

**Affiliations:** 1 Department of Health Economics, Center for Public Health, Medical University of Vienna, Kinderspitalgasse 15, 1090 Vienna, Austria; 2 Department of Psychiatry, Faculty of Medicine, University of Szeged, Korányi Alley 8-10, Szeged, 6720 Hungary; 3 Department of Preventive Medicine, Faculty of Public Health, University of Debrecen, 26 Kassai Street, Debrecen, Hungary; 4 Department of Psychiatry, Warneford Hospital, University of Oxford, Oxford, OX3 7JX UK

**Keywords:** OxCAP-MH, Translation, Cultural and linguistic adaptation, Hungarian, Mental health, Capability approach, Freedom of choice, Well-being, Quality of life

## Abstract

**Background:**

A link between mental health and freedom of choice has long been established, in fact, the loss of freedom of choice is one of the possible defining features of mental disorders. Freedom of choice has internal and external aspects explicitly identified within the capability approach, but received little explicit attention in capability instruments. This study aimed to develop a feasible and linguistically and culturally appropriate Hungarian version of the Oxford CAPabilities questionnaire—Mental Health (OxCAP-MH) for mental health outcome measurement.

**Methods:**

Following forward and back translations, a reconciled Hungarian version of the OxCAP-MH was developed following professional consensus guidelines of the International Society for Pharmacoeconomics and Outcomes Research and the WHO. The wording of the questionnaire underwent cultural and linguistic validation through content analysis of cognitive debriefing interviews with 11 Hungarian speaking mental health patients in 2019. Results were compared with those from the development of the German version and the original English version with special focus on linguistic aspects.

**Results:**

Twenty-nine phrases were translated. There were linguistic differences in each question and answer options due to the high number of inflected, affixed words and word fragments that characterize the Hungarian language in general. Major linguistic differences were also revealed between the internal and external aspects of capability freedom of choices which appear much more explicit in the Hungarian than in the English or German languages. A re-analysis of the capability freedom of choice concepts in the existing language versions exposed the need for minor amendments also in the English version in order to allow the development of future culturally, linguistically and conceptually valid translations.

**Conclusion:**

The internal and external freedom of choice impacts of mental health conditions require different care/policy measures. Their explicit consideration is necessary for the conceptually harmonised operationalisation of the capability approach for (mental) health outcome measurement in diverse cultural and linguistic contexts.

**Supplementary Information:**

The online version contains supplementary material available at 10.1186/s40359-021-00660-0.

## Background

The capability framework was originally developed by Amartya Sen with a core focus on what individuals are free and able to do (i.e., capable of) [1]. The capability approach acknowledges that economic and social arrangements should be evaluated in terms of the freedoms enjoyed by those who live in them [2]. Sen proposes that freedom has two, sometimes overlapping aspects, including the “*processes that allow freedom of actions and decisions, and the actual opportunities that people have, given their personal and social circumstances*” [3] (p. 17). The processes that enable things to happen are rather external features, whilst the opportunity aspect has a more internal implication and “*is concerned primarily with our ability to achieve, rather than with the process through which that achievement comes about*” [4] (p. 585).

Recent literature reviews [5–7] demonstrated the growing interest in the application of the capability approach and the development of several capability instruments for the assessment of health and social care interventions. However, the differential aspects of freedom of choice have not been extensively investigated in the area of health research so far. Mental health research is an important field for the application of the capability approach because of the need to reduce inequalities across groups, reinforce patient participation in social activities, and incur improvements in how a person can live their life beyond more narrow health improvement outcomes [8]. A link between mental health and freedom of choice has long been established, in fact, the loss of freedom of choice is one of the possible defining features of mental disorders [9]. Mental health research also acknowledges a distinction between two different aspects of freedom of choice, and interprets freedom of choice as a concept, which arises if an individual is able to employ certain abilities and processes to re-determine both external and internal stimuli [10]. Mental disorders typically influence the internal freedom of choice of patients. However, external constraints can become the basis of internal restrictions (e.g. compulsion in certain life circumstances), and internal constraints caused by mental disorders can be less significant if counterbalanced by adequate support or circumstances [11]. Good examples of the latter one are addictions and phobias, where internal freedom capacity is restricted, but could be improved by external support or restrictions.

Mental health research recognizes the importance of freedom of choice because different mental disorders can affect the free will of patients to a different degree [9]. The quantification of this effect enables a better and broader measurement and valuation of impacts of mental health interventions and more relevant information for health services/policy making. So far there are two capability instruments which have already been used in the area of mental health [5]. The ICEpop CAPability measure for Adults (ICECAP-A) is a measure of capability for the general adult (18+) population [12]. Its five items include attachment, stability, achievement, enjoyment and autonomy. The ICECAP-A has been validated in the area of depression [13]; but it has not yet been used in other aspects of mental health. The Oxford CAPabilities questionnaire—Mental Health (OxCAP-MH), which was purposively built for the mental health context, is a 16-item index measure including: daily activities; social networks; losing sleep over worry; enjoying social and recreational activities; having suitable accommodation; feeling safe; likelihood of assault; likelihood of discrimination; influencing local decisions; freedom of expression; appreciation of nature; respecting and valuing people; friendship and support; self-determination; imagination and creativity, and access to interesting activities [14]. Good psychometric properties of the English [15] and German [16–18] versions of the OxCAP-MH have already been demonstrated.

Developing a culturally and linguistically appropriate version of a questionnaire in an additional language is a useful step towards a deeper understanding of the construct in a cross-cultural context [19, 20]. Freedom of choice bears varying importance for individuals in dissimilar societies and is associated with different concepts, including individuality, rationality or law [21]. Hence, the different concepts of freedom of choice may be expressed diversely in different languages. Translations of questionnaires are typically influenced by three potential issues: ambiguity, interference caused by diverse cultural backgrounds and lack of equivalence [20]. The linguistic and cultural validity of the OxCAP-MH has only been tested between the English and German languages so far. The cognitive debriefing study conducted for the German version confirmed its feasibility, but also identified some issues, which resulted in relevant changes of the text [22]. These issues were mainly related to cross-country and regional variances in the German language and differences in political and social systems. Generally, equivalent words and expressions could be found to be part of the text, which could be explained by the fact that both the English and German languages belong to the West Germanic language family [23]. Translating the OxCAP-MH questionnaire to a language that belongs to a different language family could depict more conceptual issues and shed light on how appropriate this questionnaire is to capture the different aspects of freedom of choice experienced by mental health patients. Furthermore, the translation of the OxCAP-MH questionnaire to further languages would provide strong evidence on the appropriate process of cross-cultural adaptation and how much equivalency between source and target based on content could be achieved, particularly related to the concept of freedom of choice. This research was driven by the idea of contributing to the development of a linguistically and culturally valid version of the OxCAP-MH in an Eastern European setting and investigating the different aspects of freedom of choice captured by the alternative language versions of the questionnaire.

The countries of Eastern Europe have gone through social and economic transition during the last three decades. Suicide rates have been high in large parts of Eastern Europe, with Hungary reporting some of the highest figures and having the highest suicide rates in the world between 1960 and 2000 [24–26]. Some authors (e.g. [25]) suggest that a high prevalence of affective disorders in the Hungarian population may be one of the most important contributors to the markedly high suicide rate of Hungary. The 2017 Mental Health ATLAS found that the burden of mental health disorders in Hungary reached 4.542 disability-adjusted life years (DALYs) lost per 100.000 population, which is among the highest figures in the world [27]. The proportion of global burden of disease accounted for by neuropsychiatric disorders is 24.7% in Hungary, which is above the average (21.22%) of Eastern European countries [28]. According to a recent survey, the proportion of severely depressed population was 7.2% in Hungary; and 11.8% struggled from severe anxiety [29]. The Hungarian version of the OxCAP-MH questionnaire was developed in this context to support future outcomes research and clinical trials in the area of mental health.

The Hungarian language is a member of the Finno-Ugric group of the Uralic language family, with many of its phrases borrowed from the surrounding Eastern European languages [30]. The Hungarian language has no similarities in its phonology or grammar with West Germanic languages. Therefore, it was expected that while the translation from English to German was based on expressions closer in their meaning, the translation from English to Hungarian would be rather different. In addition, the Hungarian language is the primary language of only one country, which is significantly smaller in size than Germany and most English-speaking countries, hence the Hungarian language is characterized by less regional differences. This posed the question whether some of the issues identified in the German translation process would be encountered in the Hungarian translation as well, including regional differences, alternative political and social systems, and politically unacceptable expressions.

Beside the primary purpose of this study to develop a feasible and linguistically and culturally appropriate Hungarian version of the OxCAP-MH capability well-being questionnaire, it also sheds light on the linguistic and cultural aspects of differential freedom of choice concepts and expressions within the application of the capability approach.

## Methods

**Fig. 1:**
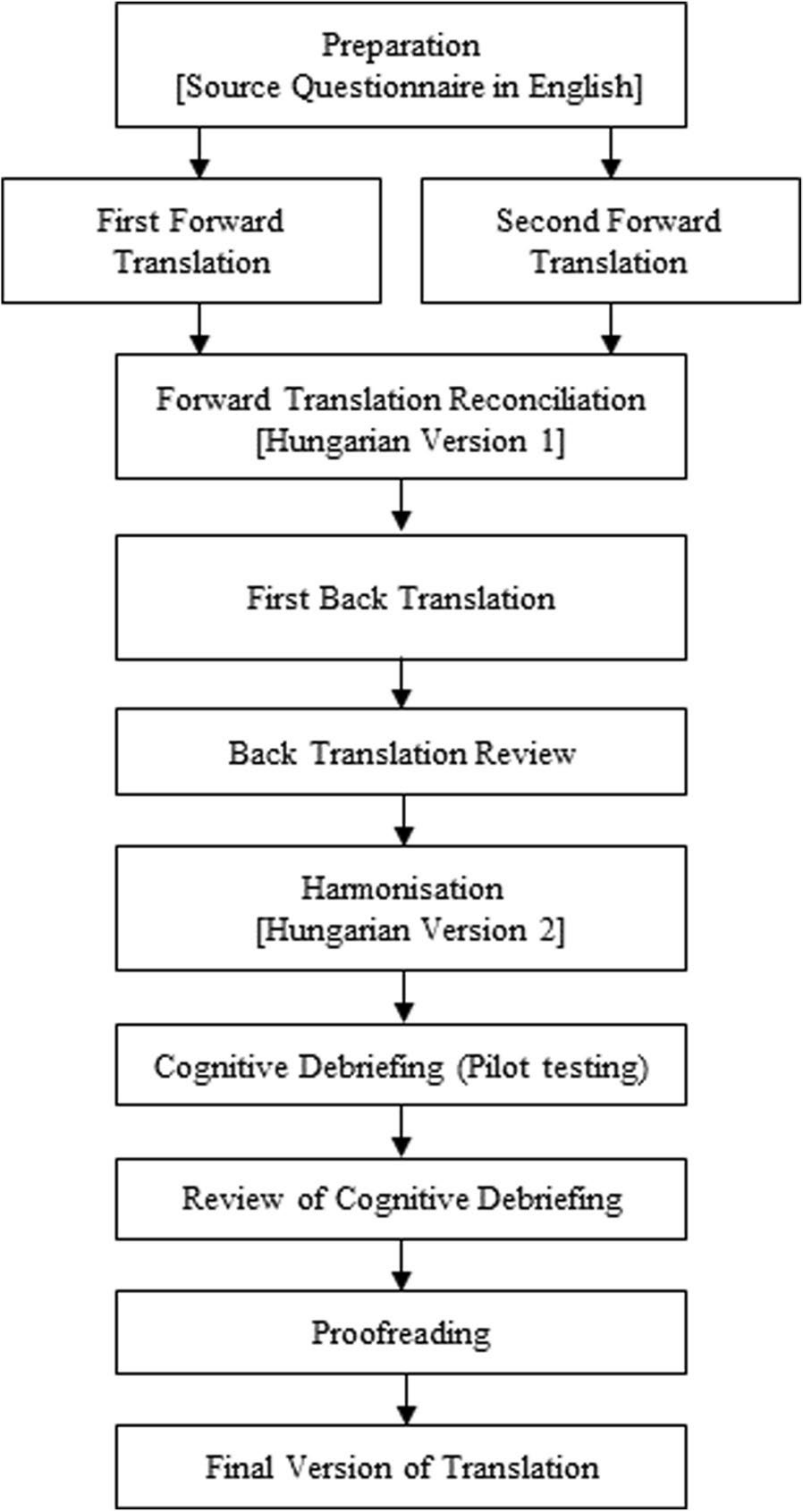
Translation process of OxCAP-MH from English to Hungarian language

The translation process followed the ISPOR [31] and WHO translation guidelines [32]. The methods were also based on those applied during the development of the German version of the OxCAP-MH instrument [16]. The full process is demonstrated in Fig. 1.

### Preparation

The preparation included some administrative steps related to the review of experiences gained during the development of the German version, permission to use the OxCAP-MH, the appointment of the key in-country persons (JK, AK), and the involvement of the translators (IK, OV) and collaborative partners (AK, IK) in the cognitive debriefing study. An explanation of concepts for the English language version of OxCAP-MH was provided by the developers (JS). This study had the unique advantages that the principal investigator (JS) is one of the instrument developers and both she and the project researcher (TH) are fluent in English, German and Hungarian languages. Not only could they evaluate the results of forward and backward translations and cognitive debriefing, but they could also compare and contrast the experiences gained during the development of OxCAP-MH in the three languages. Overall, the panel had expertise in medical translation, outcomes research, health economics, health services research, psychiatry, and public health.

### Forward translations

Two independent and qualified translators (IK, OV) carried out the forward translation from English to Hungarian language in March 2019. Both translators are native Hungarian speakers with proficiency in English, specialized or experienced in medical translations and had a minimum of three years of experience. The two independent Hungarian versions of the questionnaire were reconciled into a single forward translation by the study team. Following good practice guidelines on reconciliation [31], the final version (Hungarian Version 1) was decided in agreement with the coordinating team and the two forward translators.

### Backward translation

One backward translation was produced by an English native speaker with a high level of understanding of the Hungarian language. The coordination team reviewed the back translation of OxCAP-MH against the “Hungarian Version 1” to identify any discrepancies, discuss any conceptual problematic issues and refine the translation. Minor changes were implemented in the Hungarian version of the OxCAP-MH and a Hungarian version 2 was developed.

### Cognitive debriefing

Cognitive debriefing aimed to confirm whether the translations were accurately understood against the intended meaning of the original English OxCAP-MH questionnaire. The “Hungarian version 2” of the instrument was tested for cognitive equivalence with a group of 11 psychiatric patients at the University of Szeged in Hungary. Ethics approval was granted by the Human Investigation Review Board, University of Szeged (ethical approval number: 22835-2/2019/EKU).

The study participants were approached by their psychiatrists (AK, IK) in the respective institutionalised settings. To be included in the study, patients had to be native Hungarian speakers, aged between 18 and 80 years old, with the ability and willingness to give written consent, and not in an active phase of their mental condition. All participants received oral and written information on the study and were asked to give informed written consent prior to the face-to-face interview. AK and IK conducted the interviews following written guidelines provided by the coordinating team.

Similar to the German translation of the OxCAP-MH [16], patients were first asked to complete the translated questionnaire alone. Secondly, each item of the questionnaire was read aloud by the interviewers and patients were asked to describe in their own words what the wording meant to them. Participants were particularly asked to comment on any wording that was difficult for them to understand and if applicable, suggest alternative wording. All interviews were recorded, transcribed and analysed by TH in Hungarian language qualitatively using a modified version of the content analysis approach [33]. This approach was selected because it involves the examination of patterns in communication in a replicable and systematic manner [34]. Internal and external aspects of freedom of choice and the themes identified in the German translation of OxCAP-MH, including “possibilities for differential interpretations”, “politically unacceptable expressions”, “cross-country language differences” and “differences in political and social systems”, were used as initial codes in the qualitative analysis. If further themes or topics emerged in the text, there was an option to include them in the analysis. Additionally, the proportion of patients were calculated, whose description of each OxCAP-MH item closely corresponded to the intended meaning of the English OxCAP-MH’s concept elaboration.

### Finalization of the questionnaire translation

The coordinating team reviewed the results of the cognitive debriefing and identified translation modifications necessary for improvement [31]. One of the main foci of this step was the clarification of how different aspects of freedom of choice may be expressed in the final Hungarian version of the questionnaire.

## Results

### Forward translation

Twenty-nine phrases were translated from the English source questionnaire to Hungarian language, including the 16 main questions of the OxCAP-MH instrument, two additional phrases not included in the final score, four instruction phrases (e.g. “Please tick one”), six different response options, and one explanatory sentence, i.e. “This questionnaire asks about your overall quality of life.”

In the two forward translations there were differences in each question but not in the answer options. This means that 17 of 23 phrases (74%) resulted in some form of disagreement. The main reason for this is that, compared with the English and German languages, the Hungarian language is a much more phonetic and agglutinative language, characterized by flexible word order. All disagreements were discussed openly and decided with the involvement of a third party (TH).

Already at this stage, it became clear that there was inconsistency in the expressions used by the two forward translators to describe the freedom of choice aspects of capabilities. In some cases, the expression “*képes vagyok*” [I am able to] appeared, which is associated with internal freedom of choice. In other instances, the expression “*lehetőségem van*” [I have the opportunity to / I am free to] was selected, which is rather associated with external freedom of choice. A reconciled single Hungarian version (“Hungarian Version 1”) of the OxCAP-MH was created based on the frequency of term usage in everyday language. In most cases, the reconciliation resulted in choosing the phrase closer to the external freedom of choice concept. This was done in full agreement of the coordinating team. A summary of the most important changes is presented in Table 1.

**Table 1:** Translation of the 16 items of the OxCAP-MH questionnaire

Q	Original English version	Major discrepancies between the two forward translations	Major changes in the reconciliation phase (involving questionnaire developer)	Back translation	Changes implemented after back translation	Hungarian translation VS 2
**1**	Does your health in any way limit your daily activities, compared to most people of your age?	“similar age” versus “same age”; “health” versus “health status”	“in any way” was changed to “how often”; Opted for “same age” and “health status”	Compared to other people of the same age, how often does your health condition limit you in any way in your daily activities?	No	Azonos korúakkal összehasonlítva, milyen gyakran korlátozza Önt az egészségi állapota bármilyen módon a mindennapi tevékenységeiben?
**–**	Always, Most of the time, Some of the time, Hardly ever, Never			Always, Often, Sometimes, Rarely, Never		Mindig, Gyakran, Néha, Ritkán, Soha
**2**	Are you able to meet socially with friends or relatives?	“able to” versus “have the opportunity”; “socialize” versus “participate in meetings”	Opted for “have the opportunity” and “socialize”	How often do you have the opportunity to socialize with your friends or relatives?	No	Milyen gyakran van lehetősége barátokkal vagy rokonokkal társasági életet élni?
**3**	In the past 4 weeks, how often have you lost sleep over worry?	“losing sleep” versus “not sleeping enough”; “worry” versus “being anxious”	Opted for “problems sleeping” and “worry”	In the past 4 weeks, how often did it happen that you were worried and had problems sleeping because of that?	No	Az elmúlt 4 hét során milyen gyakran fordult elő, hogy aggódott, és ebből kifolyólag alvási problémái voltak?
**4**	In the past 4 weeks, how often have you been able to enjoy your recreational activities?	“enjoy” versus “experience pleasure”; “how much” versus “how often”	Opted for “experience pleasure” and “how often”	How often during the past 4 weeks did you have the opportunity to experience pleasure in a free-time activity?	No	Milyen gyakran volt lehetősége az elmúlt 4 hét során örömét lelni szabadidős tevékenységekben?
**5**	How suitable or unsuitable is your accommodation for your current needs?	“suitable” versus “appropriate”; “accommodation” versus “place of residence”	Opted for “suitable” and “place of residence”	How able or unable is your place of residence to satisfy your current needs?	No	Mennyire alkalmas vagy alkalmatlan a lakóhelye az Ön jelenlegi igényei kielégítésére?
**–**	Very suitable, Fairly suitable, Neither suitable nor unsuitable, Fairly unsuitable, Very unsuitable			Very able, Rather able, Neither able nor unable, Rather unable, Very unable		Nagyon alkalmas, Meglehetősen alkalmas, Se nem alkalmas, se nem alkalmatlan, Meglehetősen alkalmatlan, Nagyon alkalmatlan
**6**	Please indicate how safe you feel walking alone in the area near your home:	“feel safe” versus “feel that you are safe”; “home” versus “place of residence”	Opted for “feel safe” and “place of residence”	Please mark how safe you feel walking around near your place of residence:	No	Kérjük, jelölje meg, hogy mennyire érzi biztonságosnak egyedül sétálni a lakhelye közelében:
**–**	Very safe, Fairly safe, Neither safe nor unsafe, Fairly safe, Very unsafe			Very safe, Rather safe, Neither safe nor unsafe, Rather unsafe, Very unsafe		Nagyon biztonságos, Meglehetősen biztonságos, Se nem biztonságos, se nem veszélyes, Meglehetősen veszélyes, Nagyon veszélyes
**7**	Please indicate how likely you believe it to be that you will be assaulted in the future (including sexual and domestic assault):	“assault” versus “attack”	Opted for “assault”	Please mark how likely you think it is that you will be assaulted in the future (including sexual violence or violence in the family):	No	Kérjük, jelölje meg, hogy mennyire tartja valószínűnek, hogy a jövőben bántalmazni fogják (beleértve a szexuális és a családon belüli erőszakot is):
**–**	Very likely, Fairly likely, Neither likely nor unlikely, Fairly unlikely, Very unlikely			Very likely, Rather likely, Neither likely nor unlikely, Rather unlikely, Very unlikely		Nagyon valószínű, Meglehetősen valószínű, Se nem valószínű, se nem valószínűtlen, Meglehetősen valószínűtlen, Nagyon valószínűtlen
**8**	How likely do you think it is that you will experience discrimination?	“how likely it is” versus “how likely you think it is”	Opted for “how likely you think it is”	How likely do you think it is that you will face discrimination?	No	Mennyire tartja valószínűnek, hogy hátrányos megkülönböztetés fogja érni?
**8a**	On what grounds do you think it is likely that you will be discriminated against?	“for which reasons” versus “what are the likely reasons”	Opted for “what are the likely reasons”	What are the likely reasons because of which you think you might face discrimination in the future?	No	Milyen okokat tart valószínűnek, amelyek miatt a jövőben hátrányos megkülönböztetés érheti?
**–**	Race/ethnicity, Gender, Religion, Sexual orientation, Age, Health or disability (incl. mental health)			Race/ethnicity, Gender, Religion, Sexual orientation, Age, Health condition or disability (including mental health)		Faj / etnikai hovatartozás, Nem, Vallás, Szexuális irányultság, Életkor, Egészségi állapot vagy fogyatékosság (beleértve a mentális egészséget is)
**9a**	I am able to influence decisions affecting my local area	“able to” versus “have the opportunity”	Opted for “have the opportunity”	I have the opportunity to influence decisions that affect my environment	Yes (changed back to “able to”)	Képes vagyok befolyásolni a lakókörnyezetemet érintő döntéseket
**–**	Strongly agree, Agree, Neither agree nor disagree, Disagree, Strongly disagree			I definitely agree, I agree, I both agree and disagree, I disagree, I definitely disagree		Határozottan egyetértek, Egyetértek, Egyet is értek meg nem is, Nem értek egyet, Határozottan nem értek egyet
**9b**	I am free to express my views, including political and religious views	“can” versus “have the opportunity”	Opted for “have the opportunity”	I have the opportunity to express my views freely, including my political or religious views	No	Lehetőségem van szabadon kifejezni a nézeteimet, beleértve a politikai és vallási nézeteimet is
**9c**	I am able to appreciate and value plants, animals and the world of nature	“able to” versus “have the opportunity”; “plants/animals” versus “world of plants/animals”	Opted for “have the opportunity” and “world of plants/animals”	I have the opportunity to appreciate the world of plants, animals and nature	Yes (changed to “able to”)	Képes vagyok értékelni a növényvilágot, az állatvilágot, illetve a természetet
**9d**	I am able to respect, value and appreciate people around me	whether to include “able to”; “around me” versus “in contact with”	Did not include “able to”; Opted for “in contact with”	I respect, appreciate and acknowledge people I am in contact with	Yes (included “able to” and changed to “around me”)	Képes vagyok tisztelni, értékelni és elismerni az engem körülvevő embereket
**9e**	I find it easy to enjoy the love, care and support of my family and/or friends	“find it easy” versus “it is not difficult”	Opted for “it is not difficult”	It is not difficult to enjoy the love, care and support from my family and/or friends	No	Nem jelent nehézséget a családom és/vagy a barátaim szeretetét, gondoskodását és támogatását élvezni
**9f**	I am free to decide for myself how to live my life	“free to” versus “have the opportunity to decide freely”	Opted for “have the opportunity to decide freely”	I have the opportunity to decide freely how I live my life	No	Lehetőségem van szabadon eldönteni, hogyan élem az életemet
**9g**	I am free to use my imagination and to express myself creatively (e.g. through art, literature, music, etc.)	“free to” versus “have the opportunity to use … freely”	Opted for “have the opportunity to use … freely”	I have the opportunity to use my imagination freely and to express myself in a creative way (e.g. with the help of art, literature, music, etc.)	No	Lehetőségem van a képzelőerőmet szabadon használni és magamat kreatív módon kifejezni (pl.: művészet, irodalom, zene stb. segítségével)
**9h**	I have access to interesting forms of activity (or employment)	“have access” versus “have the opportunity”	Opted for “have the opportunity”	I have the opportunity to participate in interesting events (or work)	No	Lehetőségem van részt venni érdekes tevékenységekben (vagy munkában)

### Backward translation

The back translation process highlighted further the linguistic differences of how capability freedom of choice is expressed in the English and Hungarian languages. As a result, relevant phrases were changed in three items in the Hungarian version 2 of OxCAP-MH with full agreement of the coordinating team.

A further major discrepancy was identified with the phrase “people around me” because the backward translation resulted in the phrase “*people I am in contact with*”. Based on the concept elaboration document of the original English OxCAP-MH, this item should focus on people surrounding the respondent, and the concept of being in contact with someone was deemed misleading. Since one of the forward translators has already brought up this issue, the text was changed to the mirrored translation of “*people around me*” (Table 1).

### Cognitive debriefing

The translated version of the OxCAP-MH (Hungarian Version 2) was pilot tested with a heterogeneous sample of 11 mental health patients in the Department of Psychiatry, University of Szeged (Table 2).

**Table 2:** Characteristics of the cognitive debriefing sample

Patient ID	Age (range)	Time (min.)	Primary diagnosis
010	70–74	26	Schizophrenia, schizotypal and delusional disorders
011	25–29	21	Mood [affective] disorders
012	65–69	23	Neurotic, stress-related and somatoform disorders
013	40–44	30	Neurotic, stress-related and somatoform disorders
014	40–44	25	Schizophrenia, schizotypal and delusional disorders
015	60–64	23	Mood [affective] disorders
016	20–24	22	Neurotic, stress-related and somatoform disorders
017	50–54	16	Mental and behavioural disorders due to use of psychoactive substances
018	30–34	27	Schizophrenia, schizotypal and delusional disorders
019	45–49	32	Schizophrenia, schizotypal and delusional disorders
020	40–44	31	Schizophrenia, schizotypal and delusional disorders

Five women and six men participated in the cognitive debriefing sessions of the Hungarian OxCAP-MH questionnaire. The mean age of study participants was 46 years (SD: 16.23; range 22–74 years). The most common diagnosis was schizophrenia, schizotypal and delusional disorders (n = 5), and all respondents had at least compulsory education to allow sufficient reading skills. The average duration of the interviews including both the times for completion and cognitive debriefing was 26 min (SD: 4.78; range 16–32 min). None of the patients experienced any major difficulties with understanding the individual item concepts or answering them. Only one patient refused to answer some of the questions due to its perceived sensitive aspect; however, this decision was deemed most likely related to the patient’s disease.

Patients summarized each item of the OxCAP-MH with their own words. The content of these statements was compared and contrasted with the original concept elaboration, which was created during the development of the English version of the OxCAP-MH instrument. A list of major and minor ambiguous terms, alternative interpretations and other discrepancies are listed in Table 3.

**Table 3:** Comparing the concept elaboration of OxCAP-MH with the interpretation of pilot study participants

Q	Original English version	Match*	Alternative interpretations and other discrepancies/ambigious terms**	Decision
**1**	Does your health in any way limit your daily activities, compared to most people of your age?	10/11	Employment discrimination due to mental health status “*I mean, for example, that if a workplace wishes to register me, then I can either hide my health status or expect it to have an impact on my employment.*”	No change
**2**	Are you able to meet socially with friends or relatives?	11/11	Patients focused on frequency of meeting “*How many times per week I could meet with friends*”	No change
**3**	In the past 4 weeks, how often have you lost sleep over worry?	10/11	Worry about sleeping problems “*How often I was worried about my sleep problems during the last 4 weeks*”	No change
**4**	In the past 4 weeks, how often have you been able to enjoy your recreational activities?	6/11	Some (n = 3) respondents missed the enjoyments aspect; “*My recreational activities include gardening, reading, going to the cinema and travelling.*”	No change
			**Some (n = 2) respondents reported something different due to the inaccurate translation of “able to”** **“*Here I gave the answer* “*never*”* because I really liked writing poetry, playing the piano, and writing music, and I had the occasion to do so. However, I still selected* “*never*” *because if there is an occasion but no idea and no inspiration, then never.*”**	Changed “have the occasion” to “able to”
**5**	How suitable or unsuitable is your accommodation for your current needs?	11/11	–	No change
**6**	Please indicate how safe you feel walking alone in the area near your home:	7/11	**Some (n = 3) respondents referred to fear of being alone and paranoias** **“*I was immediately reminded of someone being paranoid and thinking they wanted to kill or attack me on the street. Some are afraid of it.*”**	No change
			**Some (n = 2) respondents referred to traffic accidents** **“*How safe is the traffic, for instance, is there a pedestrian crossing, roundabout, whether drivers are in a hurry. Even a cyclist needs to consider how they are getting around. And also pedestrian, of course.*”**	No change
**7**	Please indicate how likely you believe it to be that you will be assaulted in the future (including sexual and domestic assault):	11/11	–	No change
**8**	How likely do you think it is that you will experience discrimination?	10/11	Question does not clarify whether mental health status is part of it “*I assumed that this question was not related to the current illness. This is why I selected the answer that it was quite unlikely.*”	No change
**8a**	On what grounds do you think it is likely that you will be discriminated against?	11/11	–	No change
**9a**	I am able to influence decisions affecting my local area	10/11	**Local area versus environment (lakókörnyezet) is ambiguous** **“*When I first read it, I was thinking of my immediate little house and my property. But now, the second time I read it, it refers to my living environment or settlement.*”**	Changed “have the occasion” to “able to”***
**9b**	I am free to express my views, including political and religious views	9/11	**Some (n = 2) respondents said that it is unclear whether it is at home or on the internet or other media** **“*Now this was again an ambiguous question, because if I mean home, yes, I can freely express my views and my religion. But if I post it on the internet, I'm not sure.*”**	No change
**9c**	I am able to appreciate and value plants, animals and the world of nature	10/11	Appreciation aspect is missing “*By this I mean whether I can distinguish between living and non-living creatures, plants and animals, or between my natural environment, and my artificial, built environment.*”	No change
**9d**	I am able to respect, value and appreciate people around me	10/11	The word “value” is closer to the meaning of “evaluate” in Hungarian “*I struggle with this word of* “*value/evaluate*”*, I do not know how to react to* “*value/evaluate*”*. So how do I* “*value/evaluate*” *this. The appreciation, the other word, is totally positive.*”	No change
**9e**	I find it easy to enjoy the love, care and support of my family and/or friends	11/11	–	No change
**9f**	I am free to decide for myself how to live my life	11/11	–	No change
**9g**	I am free to use my imagination and to express myself creatively (e.g. through art, literature, music, etc.)	8/11	**Some (n = 3) respondents focused on external opportunity rather than the internal aspect** **“*I interpreted this as saying that if I have the inspiration and the conditions are right, then I can always create something from a piece of paper or, if I have to, using the computer. There is nothing that stops me from expressing these feelings. However, they are not there at the moment…*”**	Changed “have the occasion” to “able to”
**9h**	I have access to interesting forms of activity (or employment)	9/11	Some (n = 2) respondents “*Well, what's interesting. It was a good question, anyway, because to take part in an interesting activity or work… Now, is it about what I find interesting, or what is in the public’s interest?*”	No change

*Number of patients, whose description closely corresponds to the intended meaning of the English OxCAP-MH’s concept elaboration

**Major discrepancies in bold

***Change was based on discussion with questionnaire developer, to align with internal and external aspects of freedom of choice

The cognitive debriefing has shown that the OxCAP-MH items were well understood by the Hungarian patients. The descriptions provided by the majority of participants closely corresponded to the intended meaning of the English OxCAP-MH’s concept elaboration guideline. The statement of each 11 patients could be fully matched to the original concept elaboration in case of seven of the 16 OxCAP-MH items. More than half of the patients provided a matching description in the remaining nine items. The identified discrepancies were mainly minor and based on ambiguous wording and the potential for differential interpretation. There were no new themes emerging compared to the qualitative analysis of the German cognitive debriefing study.^1^

However, the cognitive debriefing identified five major issues where a potential change had to be considered by the coordinating team. Two of these related to the fact that, as opposed to English, the Hungarian language clearly uses different expressions for external and internal freedom of choice concepts. As one of the patients phrased it regarding Question 4, “*if there is an opportunity but no idea and no inspiration, then I can never [enjoy recreational activities]*”. Another patient described Question 9b as “*I interpreted this as saying that if I have the inspiration and the conditions are right, then I can always create something from a piece of paper or, if I have to, using the computer. There is nothing that stops me from expressing these feelings. However, they are not there at the moment…*” Relevant changes were implemented in these two items corresponding to the underlying original conceptual aspects.

Further major issues arose from the differential interpretations of some widely used Hungarian terms. Three respondents interpreted the expression “feeling safe” in Question 6 as the fear of being alone and having paranoias, and two patients associated to traffic accidents. The term “local area” in Question 9a was ambiguous for one participant. Two respondents had difficulties summarizing Question 9b because they felt that it was unclear whether the question refers to the home environment or the internet or other media. The majority of the other respondents understood these statements as intended; hence, the coordinating team concluded that these discrepancies can be explained by individual misinterpretations or severity of disease conditions. The terms used for these phrases of Hungarian Version 2 are the ones closest to the intended meaning of the OxCAP-MH statements and were therefore not changed in the final version.

## Discussion

The paper describes the process of developing a linguistically and culturally appropriate Hungarian version of the OxCAP-MH. The paper is unique in showing the potential need for an iterative revision of the wording of an original capability instrument in the area of mental health. This is particularly important to allow the feasibility of conceptually harmonised instrument transfer between countries with greatly differing linguistic and cultural backgrounds. The robust linguistic and cultural adaptation methods and the new original language version can be seen as the main strengths of this paper. However, the study also provided evidence upon the importance of minor wording changes which did not differ in their original concept elaboration and allowed better transferability to more diverse cultures and languages.

The majority of issues needing reconciliation were identified in the forward translation process and during developing the Hungarian Version 1. This was different from the English to German translation experience, where most changes were implemented after the cognitive debriefing process. The primary reason for this difference is the phonetic and agglutinative nature of the Hungarian language and the more flexible word order. This means that the same content can be expressed correctly in more ways than in the English or German languages. A further explanation for this phenomenon is that the English and German languages belong to the same language family and are therefore grammatically closer to each other. The first reconciliation process therefore had more emphasis on the internal consistency within the questionnaire. The German translation process was also highly influenced by cross-country differences between Germany and Austria. The Hungarian language is not characterized by strong regional differences; hence, this issue did not play a role in the translation process. This was also true for political and social systems and expressions which would be considered politically unacceptable.

On the other hand, the study highlighted a so far neglected aspects of the application of the capability approach in mental health research, that of differential interpretations of internal and external freedom of choice. The findings of this paper are consistent with existing mental health and capability literature on separate internal and external aspects of freedom of choice. All steps in the translation process highlighted the linguistic discrepancies between the English and Hungarian languages in expressing capabilities freedom of choice, but it was not until the debriefing pilot that the relevant underlying conceptual differences between external and internal freedom of choice became apparent. The Hungarian language makes a clearer distinction between these two concepts than the English language, and the German language differentiates even less. The term “*able to*” is the most central expression of the OxCAP-MH questionnaire because capabilities are expressed through this term in different contexts. Each step of the translation process identified some items where this expression had to be adjusted to the intended meaning. Beside the linguistic differences in expressing freedom of choice in English and Hungarian languages, which further underline the need for robust methodological design in the translation process, e.g. professional back translation and a cognitive debriefing study in the target country, our study demonstrates the need for well-defined elaboration of the underlying freedom of choice concepts, when designing an instrument for the application of the capability approach in the mental health research area. The need to distinguish between internal and external aspects of freedom of choice is highlighted by the fact that they require different policy responses. Considering that these issues are not expected to be unique to the OxCAP-MH instrument, but likely to be so far overlooked aspects in the operationalisation of the capability approach, particularly in the area of mental health. The term “*able to*” is also used in the terminology of the ICECAP-A and some mental health instruments, hence, the findings of this study are generalizable to a wider audience.

In addition, this research found that the terms used for the distinction between the internal and external aspects of freedom of choice are somewhat different in Sen’s original capability approach work. Sen calls internal freedom of choice as the opportunity aspect, however, throughout this translation process, “*having the opportunity*” has developed into the expression for external freedom of choice. The reason for this probably lies in Sen’s focus on international development, whilst this research was conducted in the area of (mental) health research. Moreover, Sen’s approach acknowledges that the two aspects of freedom of choice are sometimes overlapping concepts, which might be relevant to some items of the OxCAP-MH, particularly to those, which do not include the term “*able to*”.

A potential limitation of this study is that, despite guidelines, only one back-translation was produced. While it was challenging to identify English native speakers with sufficient Hungarian knowledge and professional background, we believe that conducting only one back-translation had little impact on the overall findings of the study. A further potential limitation of this study is that nearly half of the cognitive debriefing sample consisted of patients with schizophrenia. This is a rather severe mental disorder often resulting also in minor or major reduction of cognitive abilities. The patients included in this study were all under treatment, had no major cognitive impairment. and provided their consent both to treatment and participation in this research. In addition, it may well be that the higher proportion of patients with schizophrenia in the sample significantly contributed to the identification and proper understanding of the importance of differential freedom of choice concepts.

The Hungarian OxCAP-MH is now available for use free of charge for non-commercial use and can be obtained the Department of Health Economics at the Medical University of Vienna. A larger scale study is needed to assess the full psychometric validity of the Hungarian OxCAP-MH instrument, similar to those conducted in English [15] and German [17, 18] speaking settings.

## Conclusion

The findings of this paper confirmed that the Hungarian language version of the OxCAP-MH is a linguistically and culturally appropriate instrument, which is feasible to use in practice and is ready for further validation. Compared with the development of the German version, there were more linguistic, but fewer culturally relevant changes throughout the translation process. The Hungarian language uses different expressions for external and internal freedom of choice concepts, which is not reflected in the English and German languages. Further translations of OxCAP-MH to diverse cultural and linguistic contexts could reveal other aspects of internal and external freedom of choice, and the distinctions made by different languages related to this concept. We suggest an explicit need for well-defined elaboration of the underlying freedom of choice concepts, when designing or translating an instrument for the application of the capability approach in the mental health research area.

## Supplementary Information

**Additional file 1:** Comparison of translation procedures in the German and Hungarian versions of OxCAP-MH.

## Data Availability

The datasets used and/or analysed during the current study available from the corresponding author on reasonable request. An English language version of the questionnaire can be found under the following link: https://healtheconomics.meduniwien.ac.at/fileadmin/healtheconomics/Documents/OxCAP-MH_ENGLISH_SAMPLE_May_2020.pdf.

## Abbreviations

ICECAP-A: ICEpop CAPability measure for Adults
ISPOR: International Society for Pharmacoeconomics and Outcomes Research
OxCAP-MH: Oxford CAPabilities questionnaire—Mental Health
WHO: World Health Organisation

## Footnotes

1 More details on the comparison of the development process of the German and Hungarian OxCAP-MH instruments can be found in Additional file 1.
