# Supplementary material for: Internal and external aspects of freedom of choice in mental health: cultural and linguistic adaptation of the Hungarian version of the Oxford CAPabilities questionnaire—Mental Health (OxCAP-MH)
Source: BMC Psychol. 2021 Oct 18;9:161. doi: 10.1186/s40359-021-00660-0 (PMC8524921; doi:10.1186/s40359-021-00660-0)
Supplement: Supplementary file 1 — Additional file 1: Comparison of translation procedures in the German and Hungarian versions of OxCAP-MH. [file 40359_2021_660_MOESM1_ESM.pdf]

Appendix 1: Comparison of translation procedures in the German and Hungarian versions of OxCAP-MH

	**GERMAN VERSION**	**HUNGARIAN VERSION**
METHODS		
Native speaker translators	Yes	Yes
Number of translators	Two forward, two backward	Two forward, one backward
Reconcilation and review steps	After forward and backward translation, and after cognitive debriefing	After forward and backward translation, and after cognitive debriefing
Reconcilation and review participants	Developer, project coordinator	Developer, project coordinator
Collaborating partner	Care provider for patients with mental health conditions	Psychiatric department of a hospital in the target country
Source of qualitative feedback	Both from patients and carers	Patients only
Further steps for adaptation	Feedback from mental health researchers from Germany was incorporated to account for cross-country differences	Not needed
Formal vs informal language	Formal (“Sie”)	Formal (“Ön”)
EMERGING THEMES		
Possibilities for differential interpretations	7 during back translation and 5 during cognitive debriefing	3 during back translation, 5 during cognitive debriefing and 1 during final reconciliation
Politically unacceptable expressions	1 (cognitive debriefing)	None identified
Cross-country language differences	2 during back translation and 1 during cognitive debriefing	Not relevant
Differences in political and social systems	1 (cognitive debriefing)	None identified
Additional emerging themes	None	None
